# Identification of Key Genes Associated with Overall Survival in Glioblastoma Multiforme Using TCGA RNA-Seq Expression Data

**DOI:** 10.3390/genes16070755

**Published:** 2025-06-27

**Authors:** Lilies Handayani, Denis Chegodaev, Ray Steven, Kenji Satou

**Affiliations:** 1 Graduate School of Natural Science and Technology, Kanazawa University, Kanazawa 9201192, Japan; lilies.stath@gmail.com (L.H.); dennis.chegodaev@gmail.com (D.C.); raysteven127@gmail.com (R.S.); 2 Department of Statistics, Tadulako University, Palu 94118, Indonesia; 3 Institute of Transdisciplinary Science for Innovation, Kanazawa University, Kanazawa 9201192, Japan

**Keywords:** glioblastoma multiforme, RNA-Seq, survival analysis, machine learning, deep learning, biomarkers, Cox regression, gene network analysis

## Abstract

**Background/Objectives:** Glioblastoma multiforme (GBM) is an aggressive and heterogeneous brain tumor with poor prognosis, emphasizing the need for reliable molecular biomarkers to improve patient stratification and treatment planning. This study aimed to identify key genes associated with overall survival in GBM by employing and comparing machine learning (ML) and deep learning (DL) approaches using RNA-Seq gene expression data. **Methods:** RNA-Seq expression and clinical data for primary GBM tumors were obtained from The Cancer Genome Atlas (TCGA). A univariate Cox proportional hazards regression was used to identify survival-associated genes. For survival prediction, ML-based feature selection techniques—RF, GB, SVM-RFE, RF-RFE, and PCA—were used to construct multivariate Cox models. Separately, DeepSurv, a DL-based survival model, was trained using the significant genes from the univariate analysis. Gradient-based importance scoring was applied to determine key genes from the DeepSurv model. **Results:** Univariate analysis yielded 694 survival-associated genes. The best ML-based Cox model (RF-RFE with 90% training data) achieved a c-index of 0.725. In comparison, DeepSurv demonstrated superior performance with a c-index of 0.822. The top 10 genes were identified from the DeepSurv analysis, including *CMTR1*, *GMPR*, and *PPY*. Kaplan–Meier survival curves confirmed their prognostic significance, and network analysis highlighted their roles in processes such as purine metabolism, RNA processing, and neuroendocrine signaling. **Conclusions:** This study demonstrates the effectiveness of combining ML and DL models to identify prognostic gene expression biomarkers in GBM, with DeepSurv providing higher predictive accuracy. The findings offer valuable insights into GBM biology and highlight candidate biomarkers for further validation and therapeutic development.

## 1. Introduction

Glioblastoma multiforme (GBM) is the most aggressive and fatal form of primary brain tumor, classified as grade IV astrocytoma by the World Health Organization [1,2,3,4]. GBM is characterized by rapid cellular proliferation, high intertumoral heterogeneity, and extensive infiltration into surrounding brain tissue, which severely limits the effectiveness of conventional therapies [5,6,7]. Despite advances in surgical resection, radiotherapy, and chemotherapy, the median overall survival of GBM patients remains approximately 14 to 15 months, with a five-year survival rate of less than 5% [8,9]. This highlights the urgent need to identify robust molecular biomarkers to improve prognostic accuracy and guide personalized therapeutic approaches [10,11,12]. It should be noted that the current study uses data collected prior to the 2021 WHO reclassification, which now defines GBM strictly as IDH-wildtype. Therefore, the term GBM in this manuscript may include cases that would now be reclassified as IDH-mutant astrocytoma.

Tumorigenesis in GBM involves complex molecular alterations, including gene mutations, aberrant gene expression, and dysregulated signaling pathways [3,13]. Gene expression profiling, particularly through RNA sequencing (RNA-Seq), provides insights into transcriptional changes that reflect tumor behavior and can reveal potential prognostic markers [14,15]. In particular, dysregulated expression of tumor suppressors and oncogenes identified through RNA-Seq has been associated with GBM progression and patient survival, supporting its utility in biomarker discovery [16,17].

The Cancer Genome Atlas (TCGA) provides a comprehensive and publicly accessible resource containing multidimensional molecular data across various cancer types, including GBM [18,19]. The TCGA GBM dataset includes RNA-Seq gene expression profiles along with detailed clinical information, such as patient survival time and vital status [20,21]. This data offers a valuable opportunity to explore the association between gene expression signatures and patient prognosis.

Recent developments in machine learning (ML) and deep learning (DL) have revolutionized cancer research by enabling the extraction of complex patterns from high-dimensional omics data [22]. These computational approaches can be used to identify potential biomarkers, classify tumor subtypes, and build accurate prognostic models. ML methods such as random forest, support vector machine, and gradient boosting machine are widely used for feature selection and survival prediction [23,24,25], while DL models such as autoencoders or deep neural networks can capture intricate non-linear relationships in large datasets [20,26].

In the context of GBM, integrating RNA-Seq expression data with survival analysis using ML and DL offers a promising strategy to uncover novel genes that are strongly associated with overall survival. However, due to the high dimensionality and variability inherent in gene expression data, careful feature selection and model validation are essential for building reliable and interpretable prognostic models.

In this study, we analyzed RNA-Seq-based gene expression profiles and corresponding survival data of GBM patients from the TCGA dataset. We applied a combination of statistical methods, machine learning algorithms, and deep learning approaches to identify key genes associated with patient prognosis. Our findings aim to provide insights into the transcriptional landscape of GBM and support the development of effective survival prediction models for clinical applications.

## 2. Materials and Methods

### 2.1. Data Collection

RNA-Seq expression data and corresponding clinical information for GBM were obtained from The Cancer Genome Atlas (TCGA) project through the Genomic Data Commons (GDC) data portal (https://portal.gdc.cancer.gov/) accessed on 24 June 2024. This study utilized data from Project ID of TCGA-GBM with accession number phs000178, specifically focusing on RNA-Seq-based gene expression quantification data (workflow type: STAR-Counts). A total of 162 samples were included in the analysis, comprising 157 tumor samples and 5 healthy samples. The dataset contained raw read counts across 29,128 genes, which were normalized using the TMM and voom transformation pipeline.

Since the dataset was collected prior to the release of the 2021 WHO classification of central nervous system tumors, IDH mutation status was not available for most samples and was therefore not used for stratification. As such, the term “GBM” in this study may include both IDH-wildtype and IDH-mutant cases based on earlier classification criteria. Patient demographic and clinical characteristics are summarized in Table 1.

### 2.2. Identification of Survival-Associated Genes

To determine genes significantly associated with overall survival in GBM patients, a univariate Cox proportional hazards regression analysis was performed on each gene individually using the gene expression data [27]. This model estimates the hazard ratio (HR) of each gene, quantifying its effect on the risk of death over time. Genes with statistically significant associations with patient survival (*p*-value < 0.05) were selected for downstream modeling. This approach enables the identification of potential prognostic biomarkers that may contribute to differences in survival outcomes among GBM patients. The Cox regression model is defined as

(1) $$h\left(t|X\right)=h_{0}\left(t\right)exp\left(\beta X\right)$$

where $h\left(t|X\right)$ is the hazard function at time t given covariates X, $h_{0}\left(t\right)$ is the baseline hazard, and β represents the estimated coefficients for each gene [28].

The univariate Cox regression analyses were implemented using R version 4.2.1 in RStudio. Survival analysis was performed using the survfit function from the survival package, and the log-rank *p*-values were extracted using the surv_pvalue function from the survminer package. Genes with *p*-values < 0.05 were considered significantly associated with overall survival and were retained for downstream analysis.

### 2.3. Integration of Machine Learning with Survival Analysis

#### 2.3.1. Feature Selection

To reduce the high dimensionality of the gene expression dataset and select the most informative features, multiple machine learning algorithms were employed for feature importance estimation. This step is essential to ensure that only relevant genes with predictive power are included in the survival models [29]. The random forest (RF) algorithm, an ensemble-based method, was used to estimate the importance of each gene based on the mean decrease in node impurity [24,25,30]. RF handles high-dimensional data well and is robust to overfit. Similarly, gradient boosting machine (GB) builds an ensemble of weak learners sequentially and focuses on reducing prediction errors by emphasizing difficult cases [25,31]. It provides feature importance based on how frequently and effectively a feature is used in boosting iterations. Support vector machine with recursive feature elimination (SVM-RFE) was applied as a wrapper method, where features are recursively removed based on the model’s weight coefficients until the optimal subset is reached [25]. This method is particularly effective in eliminating irrelevant genes that contribute little to classification performance. Additionally, random forest recursive feature elimination (RF-RFE), a variant of RFE using RF as the base model, was employed to iteratively remove the least important features based on RF’s internal ranking [25,29]. Lastly, principal component analysis (PCA), an unsupervised dimensionality reduction technique, was used to transform the original gene space into a smaller set of uncorrelated principal components. Though PCA does not directly rank individual genes, it helps identify key variance-explaining gene combinations which were further analyzed [29]. The top-ranked genes from each method were consolidated and used for survival modeling.

To implement these feature selection methods, all analyses were conducted using R version 4.2.1. Table 2 summarizes the methods, corresponding R packages, functions, and key parameters used for reproducibility.

#### 2.3.2. Survival Modeling Using Cox Proportional Hazards

The most informative genes identified through feature selection were used to develop multivariate Cox proportional hazards models to predict patient survival [32]. To assess the robustness and generalizability of the model, the dataset was split into training and testing subsets with varying proportions (60%, 70%, 80%, and 90% training data), and a 5-fold cross-validation strategy was implemented within the training set. This modeling approach allows for the estimation of the joint effects of multiple genes on survival [21,29]. Models were trained and optimized using only the training subset, while model performance was evaluated on the hold-out testing set [24]. The genes included in the best-performing models across different splits were considered robust survival biomarkers.

### 2.4. Integration of Deep Learning with Survival Analysis

#### 2.4.1. DeepSurv Model Development

In addition to traditional survival models, a deep learning-based survival analysis was conducted using the DeepSurv framework [33,34]. DeepSurv is a deep feedforward neural network that extends the Cox proportional hazards model by incorporating the ability to model complex and non-linear relationships among input features [20,26]. In this study, the input features for DeepSurv consisted of all genes that were identified as significant through univariate survival analysis.

The DeepSurv model was implemented in R (version 4.2.1) using the Keras and TensorFlow libraries. Multiple training–testing data splits were evaluated (60%, 70%, 80%, and 90% training), with 5-fold cross-validation applied to ensure the robustness and generalizability of the model [26]. To achieve optimal predictive performance, a comprehensive hyperparameter tuning process was employed. The hyperparameter space included number of hidden units (16, 32, 64), activation functions (“relu”, “tanh”), dropout rates (0.0, 0.1, 0.2), learning rates (10^−5^, 10^−6^, 10^−7^), number of epochs (30, 40, 50), and batch sizes (8, 12, 16). As a regularization scheme, dropout was used, and L1 or L2 regularization was not applied. For each training split, 486 model configurations were evaluated. By iteratively evaluating different combinations of these parameters through grid search and cross-validation, the configuration that yielded the best predictive performance on the validation data was selected as the final model. This approach enabled the model to effectively learn intricate patterns within the gene expression data that are potentially associated with patient survival, thereby enhancing its prognostic capabilities.

#### 2.4.2. Feature Importance Extraction from DeepSurv

After training the DeepSurv model, a gradient-based approach was employed to identify the most influential genes for survival prediction. This technique calculates the gradient of the model’s output (predicted risk) with respect to each input gene, quantifying how much a slight change in a gene’s value affects the prediction. Using TensorFlow’s GradientTape, gradients were computed across the test dataset. The absolute values of these gradients were averaged across all test samples to produce an important score for each gene [35,36]. The top genes with the highest average importance were selected as key contributors and exported for further analysis. This method provides an interpretable mechanism to extract biologically relevant features from deep learning models, which are often perceived as black boxes.

### 2.5. Model Performance Evaluation

To evaluate the performance of both traditional Cox and DeepSurv models, the concordance index (c-index) was employed. The c-index is a widely used metric in survival analysis to measure the discriminatory power of a predictive model. Formally, for a pair of individuals i and j, let $y_{i}$ and $y_{j}$ represent the observed survival times and ${\hat{y}}_{i}$ and ${\hat{y}}_{j}$ the corresponding predicted risks. The c-index estimates the probability that the model assigns a higher risk to the patient with the shorter survival time and is defined as

(2) $$C=P\left({\hat{y}}_{i}>{\hat{y}}_{j}|y_{i}<y_{j}\right)$$

The c-index value ranges from 0 to 1, where a higher value indicates better model performance. In this study, the c-index was computed on the test dataset for each model and used as the primary metric to select the best-performing model configurations and genes [20].

### 2.6. Kaplan–Meier Survival Analysis

To validate the clinical utility of the selected prognostic genes, Kaplan–Meier (KM) survival curves were plotted to visualize survival differences between patient subgroups [37]. Patients were stratified into high-risk and low-risk groups based on the median predicted risk score or gene expression level [38]. The log-rank test was applied to determine whether the observed survival differences between the groups were statistically significant [39]. A significant *p*-value supports the prognostic relevance of the gene under investigation. KM analysis provides both a statistical test and an intuitive visual representation of how gene expression profiles or risk scores influence survival probabilities over time [40], where a greater separation between curves indicates stronger predictive power.

### 2.7. Pathway and Network Analysis

To understand the biological functions and interactions of the top genes identified through machine learning and deep learning, pathway enrichment and gene interaction network analysis was conducted using GeneMANIA (https://genemania.org/) accessed on 16 May 2025. GeneMANIA integrates data from multiple sources, including co-expression, co-localization, protein–protein interactions, and shared pathways, to predict gene functions and visualize biological networks. This integrative approach enables the identification of functional modules and potential regulatory relationships among genes [41]. The resulting networks and enriched pathways provide valuable insight into the possible mechanisms by which these genes influence GBM prognosis, including their roles in metabolic regulation, signal transduction, and cellular structural dynamics [42,43].

## 3. Results

### 3.1. Clinical Data Summary

This clinical dataset includes information on survival status, overall survival duration, gender, and patient follow-up time. The majority of patients in this dataset were deceased at the end of the observation period, accounting for 80%, while 20% of patients were still alive. Gender distribution shows that approximately 65% were male and 35% were female, indicating a male-dominated patient population in this study. Among the deceased patients, overall survival duration varied widely, ranging from 5 to 1537 days, with a median value of approximately 360 days, indicating significant variability in patient survival after diagnosis or the start of observation. For the surviving patients, the follow-up duration ranged from 13 to 958 days, with a median of approximately 268 days. Overall, the variation in survival status and observation period within this patient cohort provides a strong basis for conducting further survival analyses, such as the Cox proportional hazards model, to evaluate the relationship between gene expression and patient prognosis.

### 3.2. Exploratory Analysis of Gene Expression Data

**Figure 1:**
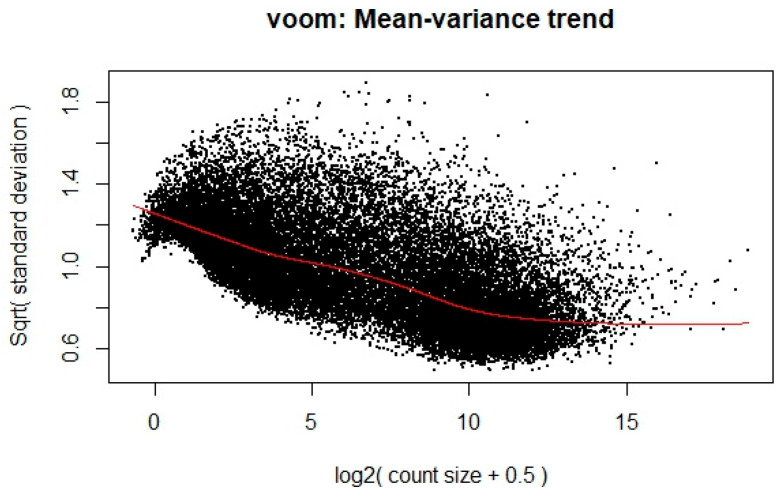
Mean variance trend plot of voom-transformed data.

To ensure the appropriateness of the RNA-Seq data for linear modeling, a voom transformation was applied to stabilize the variance across the range of gene expression levels. This transformation is crucial for meeting the assumptions of linear modeling, particularly in handling the heteroscedasticity typical of RNA-Seq data. The transformation’s effect is illustrated in Figure 1, where each dot represents a gene. The x-axis shows the average log2 counts per million, while the y-axis displays the square root of the standard deviation. A clear downward trend is observed along the red trend line, indicating that genes with lower expression levels tend to exhibit higher variability, whereas highly expressed genes show more stable variance. This trend enables the calculation of observation-level precision weights, thereby improving the reliability of downstream differential expression analysis.

**Figure 2:**
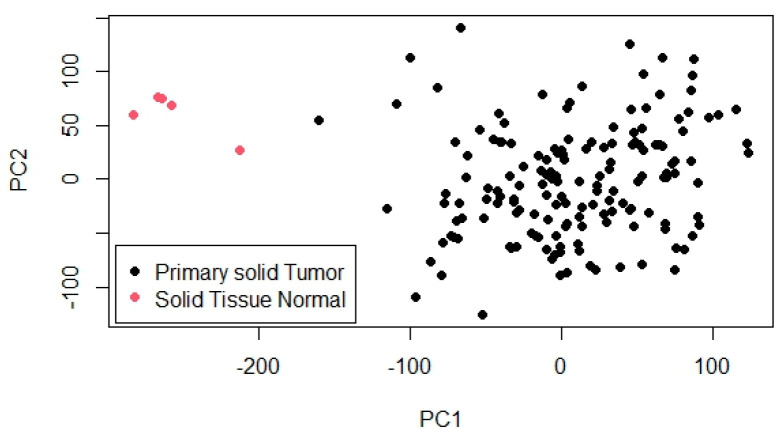
Principal component analysis of tumor and normal tissue samples.

To further explore the overall structure and variability within the dataset, PCA was conducted. The PCA results are shown in Figure 2, which visualizes the projection of samples onto the first two principal components (PC1 and PC2). In this plot, primary solid tumor samples are represented by black dots and normal solid tissue samples by pink dots. A distinct separation between the two groups is evident, particularly along PC1, which captures the largest proportion of variance and serves as the main axis distinguishing tumor from normal samples. PC2 accounts for additional, though smaller, variation. This clear clustering pattern indicates substantial differences in gene expression profiles between tumor and normal tissues, highlighting the biological relevance of the data and supporting its suitability for identifying differentially expressed genes and building prognostic models.

### 3.3. Univariate Survival Analysis Using Gene Expression Data

To assess the relationship between gene expression and overall survival, a univariate survival analysis was conducted using KM curves and log-rank tests. The gene expression matrix, consisting of 29,128 genes, was first pre-processed to match with clinical survival data. Genes with incomplete expression profiles were excluded from further analysis to ensure reliable statistical inference. For each gene, patients were stratified into two groups, high expression and low expression, based on the mean expression value of the respective gene across all samples. A KM survival model was then fitted to compare the survival distributions between these two groups, and a log-rank test was used to compute the *p*-value indicating statistical significance. This procedure was iteratively applied to all genes. Only genes with a *p*-value < 0.05 were retained as significantly associated with overall survival. As a result, 694 genes were identified as statistically significant, suggesting their potential relevance as prognostic biomarkers.

### 3.4. Machine Learning-Based Feature Selection and Survival Prediction Performance

To assess the impact of different feature selection techniques on survival prediction, multivariate Cox proportional hazards models were developed using gene sets selected by five machine learning algorithms. These models were trained and evaluated under varying training–test splits (60%, 70%, 80%, and 90% training data), and their predictive performance was measured using the c-index. The results are summarized in Table 3.

The highest predictive performance was achieved using RF-RFE at 90% training data, with a c-index of 0.725, suggesting that this method was most effective in selecting gene features relevant to survival outcomes. The substantial increase in performance at 90% training also highlights the benefit of a larger training set when dealing with high-dimensional biological data. Across all training splits, PCA consistently yielded moderate to good performance, with a notable c-index of 0.619 at the 60% split. Despite being an unsupervised method, PCA’s ability to retain variance from the original dataset may have captured essential biological signals related to survival. Meanwhile, SVM-RFE and GB also demonstrated stable performance, especially at higher training proportions. GB maintained C-index values above 0.59 across all data splits, while SVM-RFE achieved a C-index of 0.627 at the 90% split. These results affirm the potential of combining ensemble and wrapper-based methods with survival analysis for robust biomarker selection. In contrast, RF without recursive elimination showed lower and relatively flat performance across different splits, indicating that direct importance ranking alone may not sufficiently refine feature selection in this context. Overall, the findings emphasize the importance of both the choice of machine learning-based feature selection method and the size of the training dataset in determining survival prediction performance. Recursive elimination techniques, particularly those based on ensemble models, appear most suitable for high-dimensional gene expression data and should be prioritized in similar analytical pipelines.

### 3.5. Application of DeepSurv for Survival Prediction and Exploration of Significant Genes

In this study, the DeepSurv approach was employed as a deep learning model for survival analysis based on gene expression in glioblastoma patients. This model is designed to capture complex non-linear relationships between gene expression and patient mortality risk—an aspect that classical statistical methods like the Cox proportional hazards model often fail to optimally model. The primary goal of applying DeepSurv is to improve survival prediction accuracy and identify the most influential genes associated with patient prognosis. The performance of DeepSurv was evaluated by testing various model training configurations, including variations in training data proportion, number of hidden units, activation functions, dropout rates, learning rates, number of epochs, and batch sizes. The evaluation results are summarized in Table 4, which presents the c-index as the main metric for assessing survival prediction accuracy. The model trained with 90% of the data achieved the highest c-index value of 0.822, indicating excellent predictive performance. This value is significantly higher than that of the best ML-based Cox model (RF-RFE), which achieved a maximum c-index of around 0.725 using RF-RFE for feature selection and Cox proportional hazards models for survival prediction.

To further evaluate the robustness of our proposed DeepSurv model, we compared its performance with a previous study [20], which used the same TCGA GBM dataset for survival prediction. As summarized in Table 5, across different training data ratios (60%, 70%, 80%, and 90%), our model consistently achieved higher average c-index values than the reference model. For instance, at 90% training data, our model reached a c-index of 0.822 compared to 0.639 reported by Feng et al. This improvement can be attributed to the comprehensive hyperparameter tuning and model architecture optimization conducted in our study, including activation function selection, dropout regularization, and learning rate adjustment. Moreover, the exploration of multiple configurations helped mitigate overfitting and enhanced generalizability. These results suggest that with appropriate tuning and feature selection, deep learning models such as DeepSurv can significantly outperform classical or previously published models even on the same dataset, highlighting their potential utility in precision oncology. As seen, larger training datasets tend to produce relatively better c-index values on the test data. Overall, the proposed model outperformed the reference model across all configurations, confirming its robustness and superior capacity to model survival outcomes from gene expression data in glioblastoma.

Following model training, gene contributions to risk prediction was evaluated using a gradient-based method. From this, 10 key genes with the highest average scores were identified, indicating their major influence on glioblastoma patient survival predictions. These top genes are *CMTR1*, *RPL23AP42*, *TSPYL1*, *AC011287.1*, *RPL7L1P8*, *CCDC107*, *AL354743.2*, *GMPR*, *PPY*, and *MT-TL1*. To better summarize their biological functions and potential prognostic roles, the top genes identified by DeepSurv are presented in Table 6.

Among the top prognostic genes identified, *CMTR1* encodes a methyltransferase involved in mRNA cap methylation and immune regulation. Recent studies revealed that CMTR1 is overregulated in various cancers and promotes ribosomal protein gene expression, thereby enhancing tumor growth. *RPL23AP42* and *RPL7L1P8* are ribosomal pseudogenes with limited direct evidence in GBM. However, other ribosomal pseudogenes, such as *RPL4P4*, have been reported as prognostic markers and may act as competitive endogenous RNAs (ceRNAs), influencing gene expression regulation in gliomas. *TSPYL1*, a chromatin remodeling factor, is associated with neural development and may contribute to glioma progression, as identified in IDH1-associated tumor evolution studies. For *AC011287.1* and *AL354743.2*, functional annotation is scarce, but they belong to the long non-coding RNA (lncRNA) class. Other lncRNAs, such as *MEG3*, have been shown to suppress glioma cell proliferation by modulating gene expression and chromatin states. These uncharacterized lncRNAs may play similar regulatory roles. *CCDC107*, though not yet directly studied in GBM, belongs to the coiled-coil domain-containing family. *CCDC103*, a related protein, has been implicated in glioma progression and cytoskeletal organization, suggesting that *CCDC107* may also impact tumor cell motility or structure. *GMPR*, a key enzyme in purine metabolism, catalyzes the conversion of GMP to IMP. While its direct involvement in GBM is underexplored, purine metabolism has been shown to regulate DNA repair and drive therapy resistance in glioblastoma. *PPY*, a neuropeptide and member of the NPY family, is part of the broader neuroendocrine signaling system. NPY receptors and intertumoral neuropeptides are active in GBM, potentially influencing tumor behavior. *MT-TL1*, a mitochondrial tRNA for leucine, plays a critical role in mitochondrial protein synthesis. Alterations in mitochondrial DNA, including tRNA genes like *MT-TL1*, have been linked to metabolic reprogramming and mitochondrial dysfunction in GBM.

These findings indicate that DeepSurv not only enhances survival prediction accuracy but also uncovers biologically relevant genes with potential as prognostic biomarkers or therapeutic targets. Nevertheless, experimental validation is essential to confirm their mechanistic roles in glioblastoma. Furthermore, challenges such as overfitting and interpretability must be addressed to enable clinical implementation of deep learning-based survival models.

### 3.6. Kaplan–Meier Survival Analysis of Key Genes

**Figure 3:**
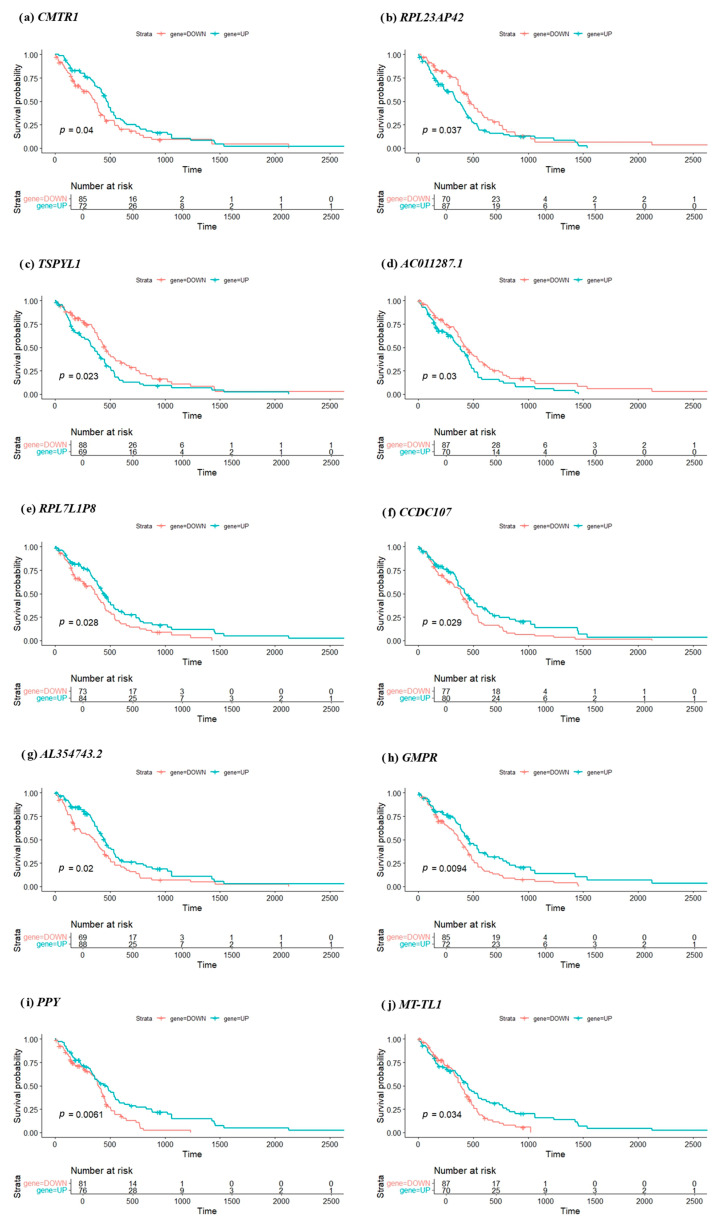
Kaplan–Meier survival curves comparing overall survival between patient groups stratified by gene expression level (UP = high expression, DOWN = low expression) for the top prognostic genes: (**a**) *CMTR1*, (**b**) *RPL23AP42*, (**c**) *TSPYL1*, (**d**) *AC011287.1*, (**e**) *RPL7L1P8*, (**f**) *CCDC107*, (**g**) *AL354743.2*, (**h**) *GMPR*, (**i**) *PPY*, (**j**) *MT-TL1*.

To evaluate the clinical relevance of the top 10 prognostic genes identified through deep learning analysis, KM survival curves were generated for each gene. Patients were stratified into high- and low-expression groups (labeled UP and DOWN) based on the median expression threshold of each gene. Survival differences between the two groups were assessed using the log-rank test. As illustrated in Figure 3, the KM plots reveal a statistically significant survival difference for all 10 genes, with log-rank test *p*-values ranging from 0.0061 to 0.04. Notably, *GMPR* (*p* = 0.0094) and *PPY* (*p* = 0.0061) exhibit the most pronounced separation between high- and low-expression groups, suggesting strong prognostic potential.

The number at risk at various time points is also shown in the plots, providing additional context for interpreting survival trends over time. These results indicate that patients with high expression (or low expression, depending on the gene) consistently have significantly different survival outcomes compared to those with opposite expression level. For example, elevated expressions of *CMTR1*, *TSPYL1*, or *RPL23AP42* are associated with poorer survival, aligning with their hypothesized roles in promoting tumor progression or interfering with gene regulation. The statistical significance of separation supports the relevance of these genes as individual prognostic biomarkers. From a clinical perspective, these genes could potentially be used to stratify glioblastoma patients into distinct risk categories, aiding personalized treatment planning. However, further investigation, including experimental validation and clinical trials, is essential before clinical implementation.

While all top 10 genes showed statistically significant survival differences, the nature of the curve separation varied. For instance, although *PPY* demonstrated the lowest *p*-value (*p* = 0.0061), its survival curves remained nearly overlapping until after the median survival time, suggesting a delayed prognostic effect. In contrast, *GMPR* (*p* = 0.0094) showed early and consistent divergence between high- and low-expression groups, indicating a more robust and clinically meaningful impact on patient prognosis. These distinctions highlight the importance of interpreting survival curves beyond *p*-values alone, considering both the magnitude and timing of risk stratification, which may influence their utility in future clinical applications.

### 3.7. Functional Network and Pathway Analysis of Key Genes

**Figure 4:**
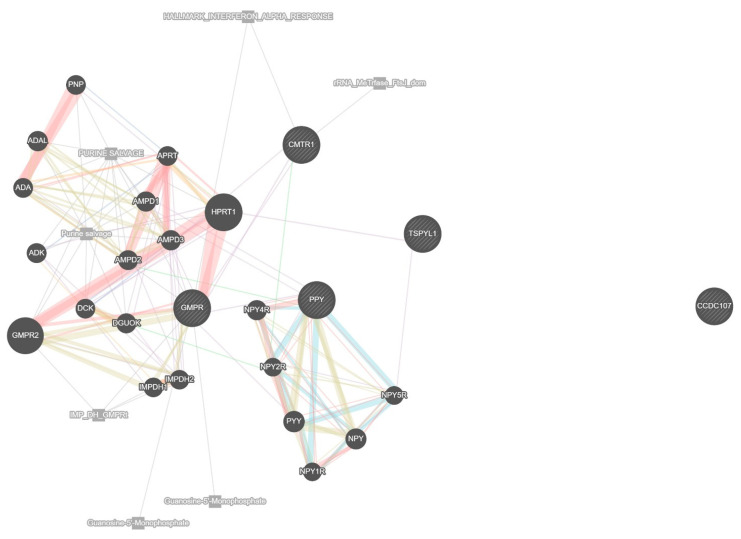
Protein–protein interaction (PPI) network highlighting hub genes among the top-ranked prognostic candidates and their interacting partners.

To further understand the biological functions and molecular interactions of the top 10 genes identified through machine learning and deep learning models, a network-based functional analysis was conducted. This analysis integrated diverse biological data sources, including co-expression, physical and genetic interactions, shared pathways, co-localization, and shared protein domains. The network was constructed for Homo sapiens, with the weighting based on biological process relevance to ensure a functionally meaningful arrangement. The resulting protein–protein interaction (PPI) network, shown in Figure 4, illustrates how the selected genes are embedded within a broader biological framework. In this network, the central 10 genes are enriched with additional interacting partners predicted.

The lines connecting the genes are color-coded to indicate the nature of their interactions; co-expression is depicted in light purple, physical interactions in pink, predicted interactions in orange, co-localization in green, shared pathways in blue, and shared protein domains in gray. These edge types reflect distinct modes of functional relationships; co-expression suggests genes are transcriptionally coordinated, physical interactions imply direct protein binding, predicted interactions are inferred from orthologous data, and co-localization indicates shared subcellular compartments. Shared pathways and shared domains reflect common biological roles or structural motifs. These visual indicators provide a comprehensive representation of the complex relationships among the genes.

The analysis reveals that many of the selected genes share strong co-expression and pathway-based associations, suggesting that they may participate in related biological processes relevant to glioblastoma progression. Notably, several genes such as *GMPR* emerge as key hubs within the network, connecting multiple genes primarily involved in purine metabolism. *GMPR* acts as a central node linking metabolic and nucleotide regulatory elements crucial for maintaining the proliferative state of cancer cells. *CMTR1*, known for its involvement in RNA processing and immune response modulation, shows limited but specific interactions, notably with *HPRT1*, suggesting a specialized regulatory role rather than widespread connectivity. Notably, *CMTR1* is not connected with *TSPYL1* in the network. *TSPYL1* exhibits limited but distinct associations, particularly with *HPRT1* and *NPY5R*, reflecting its involvement in regulatory pathways such as chromatin remodeling and cell cycle control. In this analysis, *TSPYL1* is not connected with *PPY* or *CMTR1,* suggesting distinct functional roles in the network. The neuropeptide-related genes, including *PPY* and members of the *NPY* family (e.g., *NPY*, *NPY1R*, *NPY2R*, *NPY4R*, *NPY5R*, *PYY*), form a distinct cluster characterized by strong co-expression and shared pathway interactions, indicating a potential role in neuroendocrine signaling relevant to glioblastoma. The network also includes *CCDC107*, which appears isolated with no direct interactions, implying that its role in glioblastoma remains unclear but may still be worth investigating given its potential involvement in cytoskeletal regulation. Overall, this functional network analysis emphasizes the interconnectedness of the selected prognostic genes and highlights their collective involvement in pathways that could be critical to glioblastoma survival outcomes. These findings provide not only a biological rationale for their selection as predictive biomarkers but also lay the groundwork for future investigations into targeted therapeutic strategies.

## 4. Discussion

This study demonstrates the effectiveness of both machine learning and deep learning methods in identifying prognostic biomarkers for GBM using RNA-Seq gene expression data. GBM remains one of the most lethal brain tumors, with limited improvement in patient outcomes over the last decades. Identifying molecular features that can reliably predict survival is crucial for improving risk stratification and guiding treatment decisions. The integration of computational approaches enables high-throughput and unbiased screening of potential biomarkers across the genome.

The univariate Cox regression analysis identified 694 survival-associated genes, which served as a foundation for building multivariate models. Among the ML-based approaches, the Cox model using random forest–recursive feature elimination (RF-RFE) with 90% training data achieved a concordance index (C-index) of 0.725, suggesting moderate predictive performance. These ML models offer interpretability and are less computationally intensive, making them attractive for practical applications. However, they may not fully capture complex interactions in high-dimensional data such as gene expression profiles.

In contrast, the DeepSurv model, a deep learning extension of the Cox proportional hazards model, demonstrated superior predictive power, achieving a C-index of 0.822. This underscores the strength of DL approaches in modeling non-linear and intricate relationships that are often missed by traditional or ML-based survival models. While the higher performance of DeepSurv is promising, it also introduces challenges in interpretability and requires more computational resources and expertise.

The top 10 genes identified by DeepSurv—*CMTR1*, *RPL23AP42*, *TSPYL1*, *AC011287.1*, *RPL7L1P8*, *CCDC107*, *AL354743.2*, *GMPR*, *PPY*, and *MT-TL1*—were further analyzed using Kaplan–Meier survival curves, which showed significant separation in survival outcomes between high- and low-risk groups. Several of these genes, such as *CMTR1* and *GMPR*, have known roles in RNA modification and purine metabolism, respectively, processes that are commonly dysregulated in cancer, particularly in aggressive subtypes like GBM. The *MT-TL1* gene is part of the mitochondrial genome and has been linked to metabolic regulation and energy production in cancer cells.

To further explore the biological relevance of these genes, GeneMANIA was employed to construct gene interaction networks and perform pathway enrichment analysis. The analysis revealed functional associations with RNA processing, neuroendocrine signaling, cell cycle regulation, and chromatin modification, all of which are critical in GBM pathogenesis. These findings are consistent with previous reports highlighting the role of transcriptional dysregulation in GBM progression, particularly as revealed through RNA-Seq–based gene expression profiling. The inclusion of lncRNAs and pseudogenes among the top-ranked features also suggests the involvement of non-coding regulatory mechanisms in gliomagenesis, an area that warrants further exploration.

To further substantiate the relevance of the top-ranked genes, we investigated existing literature to determine whether these genes have been previously associated with prognosis in other types of cancer. Several of them have demonstrated potential prognostic or functional roles across various tumor types. A summary of the prognostic relevance of the top 10 genes based on published studies is provided in Table 7, which strengthens the biological plausibility of their involvement in GBM outcomes and supports their consideration as candidate biomarkers for future validation.

Our findings are consistent with recent studies that emphasize the value of combining high-throughput omics data with advanced computational models for biomarker discovery. For instance, previous research using expression data has demonstrated the prognostic significance of immune infiltration, stemness features, and metabolic reprogramming in GBM. However, our approach uniquely compares ML- and DL-based survival modeling, providing deeper insight into the relative strengths, limitations, and biological relevance of selected features derived from RNA-Seq data.

Despite these promising results, this study has some limitations. First, the analysis relies solely on TCGA data, which may introduce cohort-specific biases and limit generalizability. Gene expression patterns can vary significantly across populations and platforms. Therefore, external validation using independent cohorts, such as CGGA or REMBRANDT, is essential to assess the robustness and transferability of the proposed models. In future work, we plan to validate the selected prognostic genes and trained models using these external datasets to ensure generalizability across different patient populations. Second, although DeepSurv provides improved performance, its black-box nature hinders straightforward biological interpretation. Future studies should also focus on enhancing model explainability and performing functional validation of key genes through wet-lab experiments, including CRISPR knockout and qRT-PCR assays.

In this study, univariate Cox regression identified 694 genes with *p*-values below 0.05. However, after applying false discovery rate (FDR) correction using the Benjamini–Hochberg method across all 29,128 tested genes, none remained statistically significant at FDR < 0.05. This result is not unexpected given the high dimensionality and multiple testing burden inherent in genome-wide gene expression analyses. Nevertheless, these initially filtered genes served as a preliminary feature pool for further downstream analysis using multivariate Cox models and deep learning, which helped prioritize biologically meaningful candidates. While the results should be interpreted cautiously, this workflow aligns with common practices in exploratory omics studies and sets the stage for future independent validation efforts.

Overall, this integrative analysis supports the utility of ML and DL techniques in survival prediction and biomarker identification in GBM. The identified genes have the potential to enhance current prognostic models and inform the development of targeted therapies. By combining statistical rigor, computational power, and biological interpretation, this study contributes to the ongoing effort to improve patient stratification and precision oncology in glioblastoma.

## 5. Conclusions

In this study, we integrated statistical, machine learning, and deep learning approaches to analyze TCGA RNA-Seq gene expression data and identify key genes associated with overall survival in GBM. Univariate Cox analysis revealed hundreds of genes with potential prognostic relevance, which were further refined using various feature selection techniques. Among traditional models, RF-RFE combined with Cox regression achieved a notable c-index of 0.725. However, the deep learning-based DeepSurv model outperformed all traditional approaches, achieving a c-index of 0.822 and identifying 10 key prognostic genes: *CMTR1*, *RPL23AP42*, *TSPYL1*, *AC011287.1*, *RPL7L1P8*, *CCDC107*, *AL354743.2*, *GMPR*, *PPY*, and *MT-TL1*. Several of these genes, such as CMTR1, play critical roles in mRNA cap methylation and immune response regulation, while *GMPR* and *MT-TL1* are involved in metabolic pathways essential for tumor growth. *PPY*, typically associated with neuroendocrine signaling, emerged as a significant prognostic indicator in glioblastoma. Kaplan–Meier analysis confirmed the clinical relevance of these genes, and network-based functional analysis revealed their potential interactions and pathways. These findings not only enhance our understanding of GBM biology but also provide a foundation for the development of personalized prognostic tools and targeted therapies. The identified genes could serve as candidate biomarkers for patient risk stratification and novel targets for therapeutic intervention. Further experimental validation and functional studies are warranted to confirm their roles and clinical applicability.

## Figures and Tables

**Table 1:** Patient cohort summary (*n* = 157).

Category	Subcategory	Value
Overall Survival Time	Mean (days)	415.54
	Standard Deviation (days)	384.81
Age	Mean (years)	59.57
	Standard Deviation (years)	13.51
Sex	Male	102
	Female	55
Vital Status	Deceased	126
	Alive	31
Ethnicity	Hispanic or Latino	4
	Not Hispanic or Latino	129
	Not Reported	24
Race	White	141
	Black or African American	10
	Asian	5
	Not Reported	1
Classification of Tumor	Primary	108
	Recurrence	12
	Progression	32
	Unknown	5

**Table 2:** Summary of feature selection methods, packages, functions, and parameters.

Method	R Package(s)	Function(s)	Key Parameters/Notes
RF	randomForest	randomForest()	*n*.trees = 100–1000; best ntree selected by accuracy
GB	gbm	gbm()	*n*.trees = 100–1000, interaction.depth = 3, shrinkage = 0.01, cv.folds = 5
SVM-RFE	caret, e1071	rfe() with svmFuncs	10-fold cross-validation; default svmLinear kernel
RF-RFE	caret, randomForest	rfe() with rfFuncs	10-fold cross-validation; sizes = c(10, 20, 50, 100); final model with ntree = 500
PCA	stats	prcomp()	Data centered and scaled; top 20 loadings from PC1 selected

**Table 3:** Average c-index values on test data for Cox proportional hazards models based on different machine learning feature selection methods.

Ratio of Training Data (%)	RF	GB	SVM-RFE	RF-RFE	PCA
60	0.585	0.605	0.598	0.606	0.619
70	0.586	0.610	0.580	0.574	0.610
80	0.571	0.590	0.613	0.585	0.596
90	0.598	0.618	0.627	0.725	0.608

**Table 4:** Average c-index values on test data for DeepSurv models based on different training.

Ratio of Training Data (%)	C-Index	Units	Activation	Dropout	Learning Rate	Epochs	Batch Size
60	0.677	16	tanh	0.1	10^−6^	40	8
70	0.737	64	tanh	0.2	10^−7^	30	12
80	0.733	64	relu	0	10^−7^	40	16
90	0.822	16	tanh	0.2	10^−5^	50	16

**Table 5:** Average c-index values of the proposed model and reference model using different amounts of training data.

Ratio of Training Data (%)	Proposed Model	Reference Model [20]
60	0.677	0.603
70	0.737	0.603
80	0.733	0.609
90	0.822	0.639

**Table 6:** Summary of top prognostic genes identified by DeepSurv with associated biological functions.

Gene Symbol	Gene Type	Known/Predicted Function	Biological Relevance to GBM	KM *p*-Value
*CMTR1*	Protein-coding	mRNA cap methylation, immune response	Upregulated in cancer; promotes ribosomal gene expression and growth [44]	0.040
*RPL23AP42* and *RPL7L1P8*	Pseudogene	Putative ceRNA, miRNA sponge, ribosomal pseudogene	Function unclear; may act as ceRNAs affecting gene regulation, as seen in others pseudogene [45]	0.037 and 0.027
*TSPYL1*	Protein-coding	Chromatin remodeling, transcription regulation	Associated with neural development and tumor progression [46]	0.023
*AC011287.1* and *AL354743.2*	lncRNA	Uncharacterized lncRNAs, potential epigenetic regulators	While direct evidence in GBM is lacking, other lncRNAs have been shown to influence glioma development and gene regulation [47]	0.029 and 0.020
*CCDC107*	Protein-coding	Coiled-coil domain	Related CCDC family member implicated in glioma progression and cytoskeletal regulation [48]	0.029
*GMPR*	Enzyme	Purine metabolism (GMP to IMP conversion)	Key enzyme in purine metabolism [49]; purine metabolism regulates DNA repair and therapy resistance in GBM [50]	0.009
*PPY*	Peptide hormone	Neuropeptide signaling	NPY receptors and intratumoral neuropeptides active in GBM [51]	0.006
*MT-TL1*	Mitochondrial tRNA	Mitochondrial protein synthesis	Mitochondrial tRNA involved in energy metabolism; mtDNA alterations in GBM [52]	0.034

**Table 7:** Prognostic relevance of the top 10 genes in other cancer types based on the existing literature.

Gene Symbol	Full Name	Cancer Type	Reported Prognostic Association	References
*CMTR1*	Cap Methyltransferase 1	Multiple cancers (notably basal-like breast cancer)	Upregulated CMTR1 promotes tumor growth via ribosome biogenesis and RNA metabolism; a potential therapeutic target.	[44]
		Colorectal cancer	High CMTR1 expression is linked to poor prognosis; it promotes tumor growth and immune evasion.	[53]
*RPL23AP42*	Ribosomal Protein L23a Pseudogene 42	Esophageal squamous cell carcinoma	High RPL23AP42 expression is linked to shorter overall survival, indicating its potential as a negative prognostic marker.	[54]
*TSPYL1*	Testis-Specific Y-Encoded-Like 1	Lung carcinoma	Loss of TSPYL1 promotes EMT via TGF-β signaling, indicating a role in cancer progression.	[55]
*AC011287.1*	Long non-coding RNA	Head and neck squamous cell carcinoma	High expression of AC011287.1 is associated with poor survival; identified as an independent unfavorable prognostic factor in multivariate Cox analysis.	[56]
		Hepatocellular carcinoma	High AC011287.1 expression is associated with poor survival; included in a high-risk lncRNA signature predictive of worse prognosis.	[57]
*RPL7L1P8*	Ribosomal Protein L7-Like 1 Pseudogene 8	Non-small cell lung cancer	Implicated in tumor–macrophage interaction; potential role in modulating immune microenvironment, but its direct prognostic role remains to be clarified.	[58]
*CCDC107*	Coiled-Coil Domain-Containing 107	Colorectal cancer	Downregulated in CRC and associated with poor disease-free survival, suggesting its potential as a diagnostic and prognostic biomarker.	[59]
*AL354743.2*	Long non-coding RNA	Not cancer-specific; studied in ulcerative colitis	Implicated in immune-related pathways and T-cell apoptosis; associated with prolonged inflammation. However, its direct role in cancer prognosis remains uncharacterized.	[60]
*GMPR*	Guanosine Monophosphate Reductase	Melanoma	GMPR is downregulated in invasive melanoma and functions as a tumor suppressor by reducing intracellular GTP levels, thereby inhibiting RAC1 activity, invadopodia formation, and tumor invasion.	[61,62]
		Lung adenocarcinoma	GMPR was included in a four-gene prognostic signature where higher risk scores associated with altered GMPR expression were linked to shorter overall survival.	[63]
*PPY*	Pancreatic Polypeptide	Pancreatic cancer	Blunted PPY response in pancreatic cancer–related diabetes, especially with tumors in the pancreatic head; prognostic value remains unclear.	[64]
		Pancreatic and gastrointestinal endocrine tumors	PPY overexpression is common in PP-producing tumors; useful for tumor identification, but its prognostic significance remains unclear.	[65]
*MT-TL1*	Mitochondrially Encoded tRNA Leu	Osteosarcoma	The m.3243A>G mutation in MT-TL1 (human equivalent of tRNA-Leu (UUR)) impairs complex I activity, leading to reduced tumorigenic potential, suggesting that severe mitochondrial dysfunction may suppress tumor progression.	[66]

## Data Availability

The RNA-Seq expression data and clinical data analyzed in this study were obtained from The Cancer Genome Atlas (TCGA) via the Genomic Data Commons (GDC) data portal (https://portal.gdc.cancer.gov/) accessed on 24 June 2024, Project ID: TCGA-GBM (accession number phs000178). All data used is publicly available.

## Abbreviations

The following abbreviations are used in this manuscript:

DL	Deep learning
GBM	Glioblastoma multiforme
GB	Gradient boosting machine
KM	Kaplan–Meier
ML	Machine learning
PCA	Principal component analysis
RF	Random forest
RFE	Recursive feature elimination
SVM	Support vector machine
TCGA	The Cancer Genome Atlas
